# Treatment of donor corneal tissue with immunomodulatory cytokines: a novel strategy to promote graft survival in high-risk corneal transplantation

**DOI:** 10.1038/s41598-017-01065-z

**Published:** 2017-04-20

**Authors:** Maryam Tahvildari, Parisa Emami-Naeini, Masahiro Omoto, Alireza Mashaghi, Sunil K. Chauhan, Reza Dana

**Affiliations:** Schepens Eye Research Institute and Massachusetts Eye and Ear Infirmary, Department of Ophthalmology, Harvard Medical School, Boston, MA USA

## Abstract

Antigen-presenting cells (APCs) play an important role in transplant rejection and tolerance. In high-risk corneal transplantation, where the graft bed is inflamed and vascularized, immature APCs in the donor corneal stroma quickly mature and migrate to lymphoid tissues to sensitize host T cells. In this study, using a mouse model of corneal transplantation, we investigated whether enrichment of tolerogenic APCs (tolAPCs) in donor corneas can enhance graft survival in corneal allograft recipients with inflamed graft beds. Treatment of donor corneas with interleukin-10 (IL-10) and transforming growth factor-β1 (TGFβ1) altered the phenotype and function of tissue-residing APCs. Transplantation of these tolAPC-enriched corneas decreased frequencies of interferon gamma (IFNγ)^+^ effector T cells (Teffs), as well as allosensitization in the hosts, diminished graft infiltration of CD45^+^ and CD4^+^ cells, and significantly improved corneal allograft survival compared to saline-injected controls. These data provide a novel approach for tolAPC-based immunotherapy in transplantation by direct cytokine conditioning of the donor tissue.

## Introduction

In high-risk corneal allograft recipients with inflamed and vascularized graft beds, corneal transplants have survival rates of 50% or lower even after treatment with corticosteroids. In order to improve graft survival and to bypass side effects of nonspecific immunosuppressants, it is crucial to develop strategies that induce alloantigen-specific immune tolerance. Antigen-presenting cells (APCs) are sentinels of the immune system and principal mediators of the adaptive immune response. They are known to play an important role in transplant rejection and tolerance; our laboratory has characterized different populations of resident immature bone marrow-derived cells in the corneal stroma, including dendritic cells (DCs) and macrophages^1–3^. Tissue resident APCs display an immature phenotype and have regulatory function, which is important for maintaining a non-inflammatory environment^4, 5^. We have previously shown that resident bone marrow-derived CD11b^+^ and CD11c^+^ cells in the central corneal stroma stay in a highly immature state under normal conditions, expressing very low levels of MHC-II and co-stimulatory molecules. During inflammation, e.g., allogeneic corneal transplantation, these resident APCs quickly mature and leave the graft site to migrate to lymphoid tissues where they prime host T cells^1^. Trafficking is significantly enhanced when grafts are placed onto inflamed host beds^6–11^. Interestingly, our studies have indicated that total depletion of graft-residing donor APCs does not improve transplant survival^12^, suggesting that while some donor APCs participate in host sensitization others may be involved in tolerance induction^13^.

Between the two states of immaturity and maturity of APCs is a ‘maturation-resistant’ or ‘tolerogenic’ state^14^. Regulatory macrophages and DCs that can promote tolerance have been extensively characterized^14–16^. Here, we define tolerogenic APCs (tolAPCs) as CD11c^+^MHC class II^lo^CD40^lo^CD86^lo^ cells. Tolerogenic APCs, which are also called maturation-resistant APCs, have the ability to induce T cell tolerance due to their diminished antigen presentation, production of regulatory cytokines, and generation and expansion of IL-10-producing regulatory T cells, and are powerful tools for promoting transplant survival^14, 17, 18^. A variety of pharmacological inhibitors have been developed to generate tolAPCs from their undifferentiated precursors in order to achieve transplant tolerance, such as immunomodulatory cytokines, rapamycin, dexamethasone, and Vitamin D^14^. In a recent study we have shown that *ex vivo* treatment of donor-type bone marrow-derived dendritic cells (BMDCs) with immunomodulatory cytokines (IL-10, TGFβ1) renders them tolerogenic, and when systemically transferred to corneal transplant recipients significantly improves allograft survival^19^. However, *ex vivo* treatment of cells faces significant translational hurdles, including safety and feasibility of cell preparations and their potential nonspecific immunosuppression when administered systemically. In this study, using a mouse model of corneal transplantation, we demonstrate that the donor cornea itself can be manipulated to generate tolAPCs. In our murine model of corneal transplantation we demonstrate that treatment of donor corneal buttons with IL-10 and TGFβ1 induces phenotypic and functional changes in tissue-resident APCs, rendering them tolerogenic and capable of suppressing allosensitization in high-risk allograft recipients that swiftly reject their corneal transplants.

## Results

### Treatment of donor corneas with IL-10 and TGFβ results in generation of tolerogenic APCs

**Figure 1:**
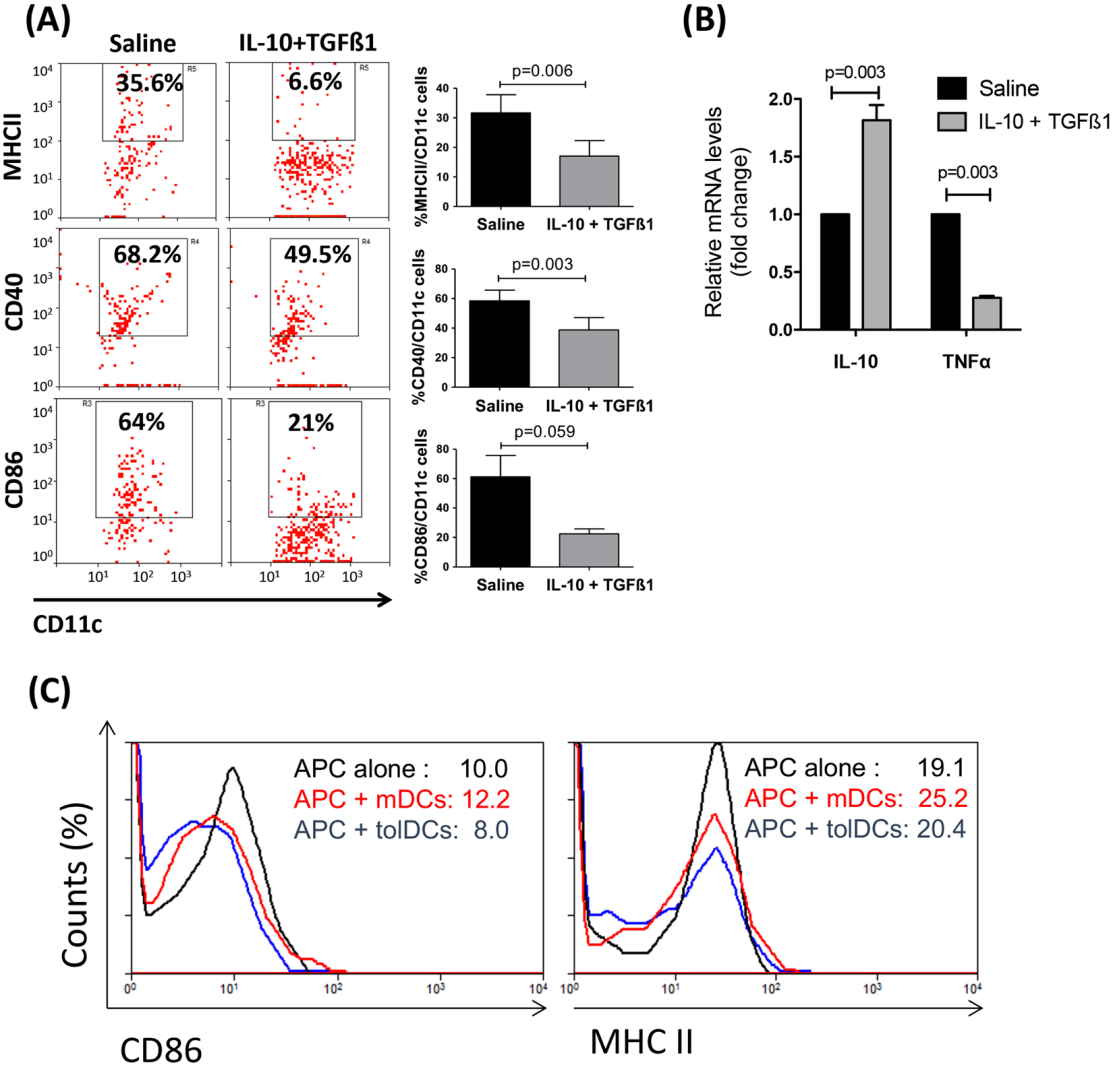
*In vivo* treatment of donor corneas with IL-10 and TGFβ1 induces tolerogenic APCs. Two days after single subconjunctival injection of IL-10 and TGFβ1, corneas were harvested and analyzed for the expression of maturation markers on dendritic cells (**A**) and mRNA expression of immunoregulatory and pro-inflammatory cytokines (**B**). (**A**) Flow cytometry analysis showing decreased frequencies of MHCII^+^CD11c^+^ cells, CD40^+^CD11c^+^ cells, and CD86^+^CD11c^+^ cells in IL-10/TGFβ1-treated corneas compared to saline-treated controls (Bar graphs demonstrate mean ± SEM; N = 6 corneas/group, data shown are representative of three independent experiments). (**B**) Real-time PCR showing mRNA expression of IL-10 and TNFα in IL-10/TGFβ1- and saline-treated corneas 48 hours after injection before transplantation. Bar graphs demonstrate mean ± SEM; p < 0.003; N = 8 corneas/group. (**C**) Bone marrow-derived dendritic cells (DCs) were treated for 5 days in the presence or absence of IL-10/TGFβ1, and stimulated with LPS overnight to obtain tolerogenic DCs (tolDCs) or mature DC (mDCs), respectively. They were then co-cultured for 24 h with allogeneic splenic APCs of GFP^+^ mice to distinguish them from bone marrow-derived DCs. Frequencies of GFP^+^ APCs expressing MHCII and CD86 were quantified by flow cytometry.

To assess whether treatment of donor ocular tissue with immunomodulatory cytokines would generate tolAPCs within the donor corneal tissue, we treated donor eyes with TGFβ1 and IL-10 *in vivo*. We performed a single subconjunctival injection of recombinant IL-10 and TGFβ1 to donor eyes, harvested corneal buttons 48 hours later and analyzed the phenotype of APCs after LPS stimulation using flow cytometry. Saline-treated control corneas showed high frequencies of mature CD11c^+^APCs, but corneas injected with IL-10 and TGFβ1 had significantly reduced frequencies of MHC II^+^ CD40^+^CD11c^+^ APCs and markedly decreased CD86^+^CD11c^+^ APCs (Fig. 1A). Real-time PCR analysis of corneas treated with IL-10 and TGFβ1 showed significantly increased IL-10, but decreased TNF-α mRNA expression compared to saline-treated corneas (Fig. 1B), suggesting a regulatory donor tissue microenvironment in the presence of such maturation-resistant tolAPCs. To determine whether tolAPCs can confer their maturation resistant phenotype to host APCs, we performed an additional *in vitro* experiment in which we co-cultured either IL-10/TGFβ1-treated bone marrow-derived dendritic cells (tolerogenic dendritic cells – tolDCs) or mature dendritic cells (mDCs) with immature host APCs. We demonstrated that host APCs acquired a tolerogenic phenotype (expressing lower levels of MHC II and CD86) when co-cultured with tolDCs but not mDCs (Fig. 1C). These findings provide evidence that tolerogenic APCs can confer maturation resistance to host APCs and can induce tolerance through blocking the indirect pathway of allosensitization.

### Induction of maturation resistant APCs in the donor cornea suppresses host allosensitization

**Figure 2:**
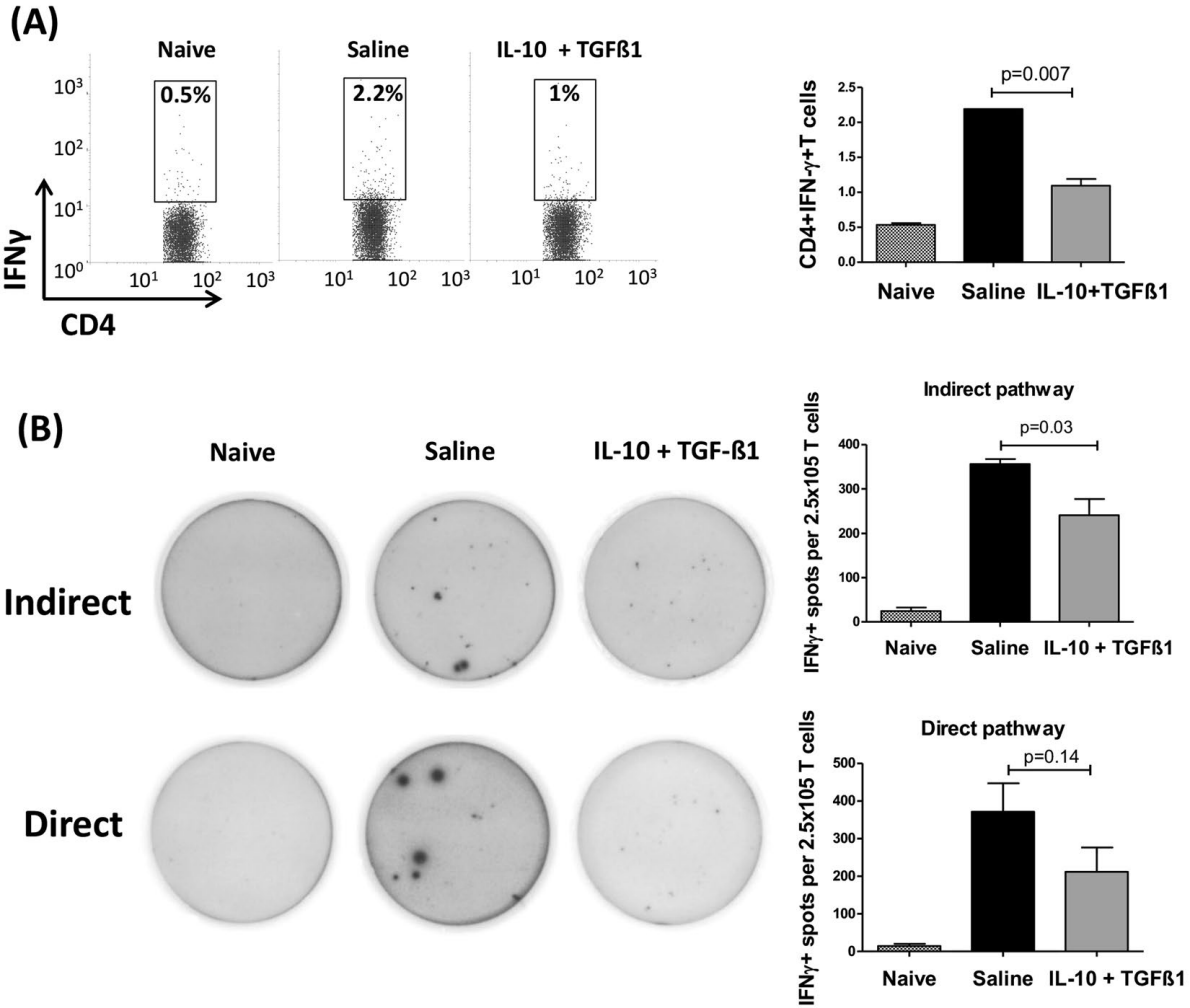
TolAPC-enriched grafts suppress allosensitization in corneal transplantation. Two days after single subconjunctival injection of IL-10 and TGFβ1 or saline, corneas were harvested and transplanted on high-risk graft beds. 2 weeks after transplantation, (**A**) frequencies of IFNγ^+^CD4^+^ Teff cells in the draining lymph nodes from naïve mice and graft recipients with IL-10/TGFβ1- and saline-treated donor corneas were analyzed using flow cytometry. Bar graphs demonstrate mean ± SEM; N = 4 mice/group, data shown are representative of two independent experiments. (**B**) For ELISPOT assay CD4^+^ T cells from draining lymph nodes of graft recipients were cultured with APCs from BALB/c (indirect pathway) or C57BL/6 mice (direct pathway) on IFNγ-coated plates. Indirectly and directly allosensitized IFNγ-producing CD4^+^ T cells were detected 48 hours later using an ELISPOT image analyzer. Bar graphs demonstrate mean ± SEM; N = 5 mice/group, data shown are representative of two independent experiments.

To assess the effect of tolAPC-enriched grafts on host allosensitization, draining lymph nodes of graft recipients with IL-10 and TGFβ1- or saline-treated corneas were harvested at 2 weeks post-transplantation to measure the frequencies of IFNγ-producing T cells using flow cytometry and the number of IFNγ^+^ allosensitized T cells using ELISPOT assay. We found that hosts with IL-10 and TGFβ1-treated donor corneas had significantly reduced Th1 cell frequencies (1.0% vs. 2.2, p = 0.007) (Fig. 2A). ELISPOT assay demonstrated significantly reduced numbers of IFNγ^+^ T cells in the indirect pathway in the graft recipients with IL-10/TGFβ1-treated donor corneas compared to controls; in addition, although not statistically significant, there was a marked decrease in the number of IFNγ^+^ T cells sensitized by the direct pathway (p = 0.14) (Fig. 2B). These data suggest that tolerogenic APCs in the donor tissue can inhibit host T cell responses.

### Treatment of donor corneas with IL-10 and TGFβ decreases graft infiltration of immune cells and increases high-risk corneal allograft survival

**Figure 3:**
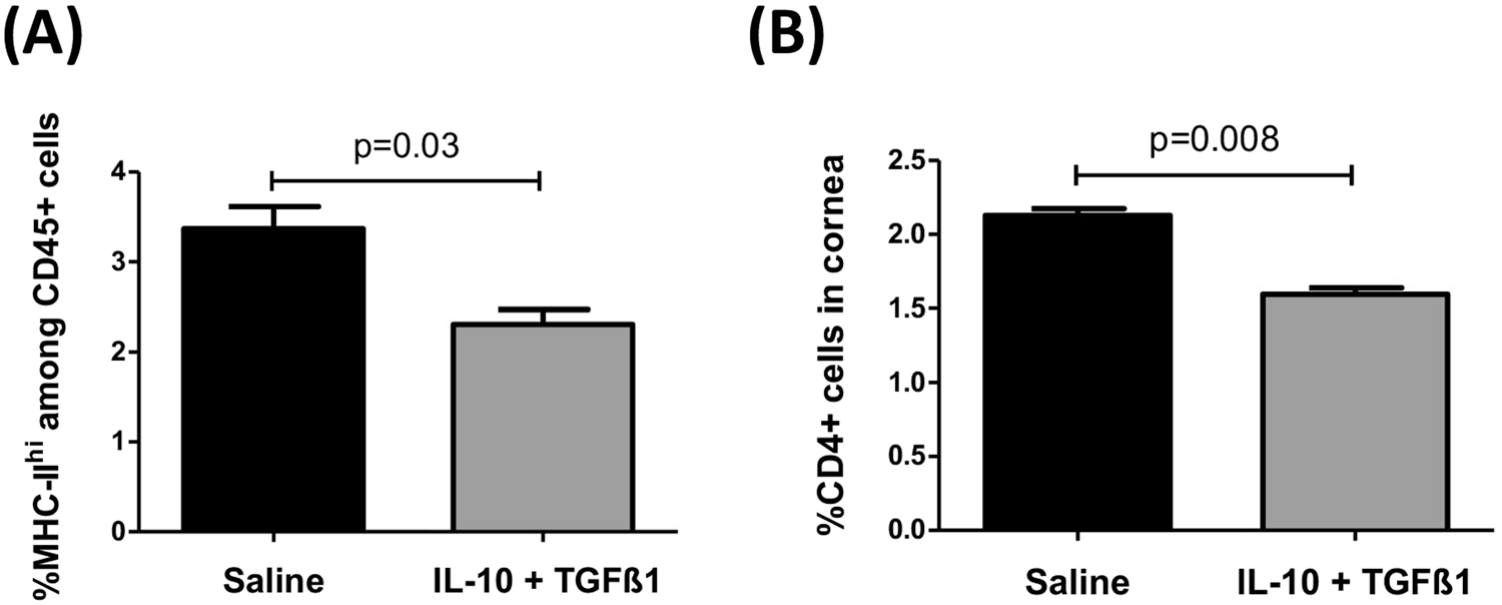
Treatment of donor corneas with IL-10 and TGFβ1 decreases infiltration of immune cells in the grafts. Two days after single subconjunctival injection of IL-10 and TGFβ1 or saline, corneas were transplanted on high-risk graft beds. Infiltration of leukocytes into the graft was determined 2 weeks after transplantation using flow cytometry. Corneal grafts were analyzed for frequencies of (**A**) MHCII^+^CD45^+^ cells and (**B**) CD4^+^ T cells. Bar graphs demonstrate mean ± SEM; N = 5 mice/group, data shown are representative of two independent experiments.

High-risk corneal transplantation promotes APC maturation, function, and migration inducing rapid allosensitization and high rates of rejection (approximately 100% in our rodent high-risk corneal transplantation model)^20^. IL-10/TGFβ1- and saline-treated corneas were transplanted on inflamed high-risk host beds. We used flow cytometry to determine whether enrichment of tolAPCs (IL-10 and TGFβ1-treated) affects corneal cell infiltration, at 2 weeks post-transplantation. Frequencies of MHCII^+^CD45^+^ cells and CD4^+^ T cells were decreased in hosts with IL-10/TGFβ1-treated donor corneas compared with controls (Fig. 3A and B).

**Figure 4:**
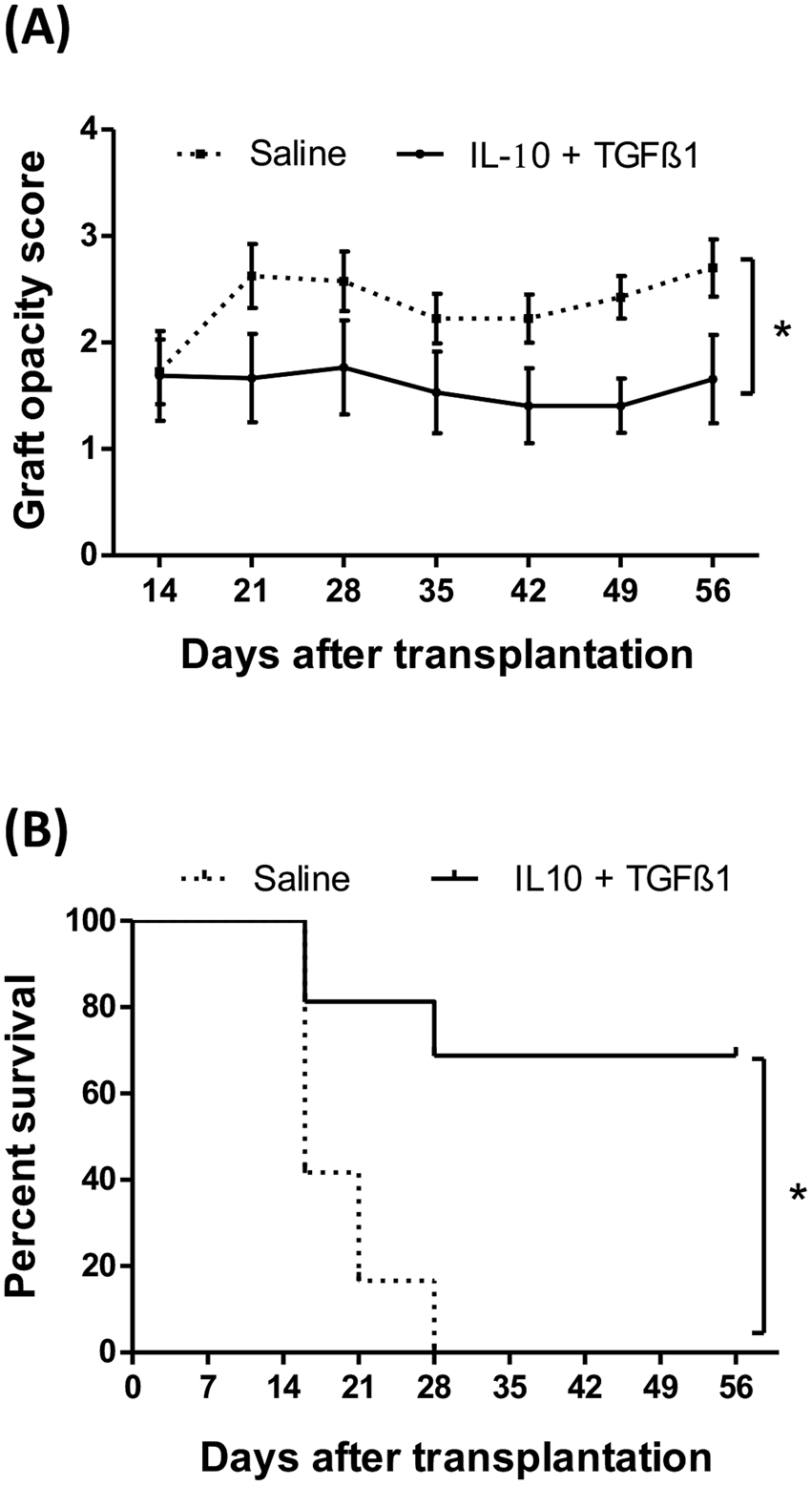
Treatment of donor corneas with IL-10 and TGFβ1 increases corneal allograft survival. Donor corneas received a single subconjunctival injection of IL-10/TGFβ1 or saline (control) two days prior to grafting. Corneal transplantation (N = 10–16/group) was performed onto inflamed graft beds. Weekly examination of grafts until 8 weeks post-transplantation revealed (**A**) decreased graft opacity scores (p = 0.0011) and (**B**) increased graft survival in IL-10/TGFβ1-treated grafts compared to saline-treated controls (68.7% vs. 0%, p < 0.0001). Error bars show standard error of mean.

To evaluate whether treatment of donor corneas with IL-10 and TGFβ1 before surgery can promote long-term allograft survival, grafts were examined weekly for up to 8 weeks post-transplantation. There was a significant decrease in corneal opacity (Fig. 4A) and a significant increase in graft survival (68.7% vs. 0%, p < 0.0001) (Fig. 4B) during weeks 3 to 8 after transplantation in IL-10/TGFβ1-treated grafts compared to saline-treated controls.

## Discussion

In this study, we show that treatment of donor corneas with IL-10 and TGFβ1 induces tolerogenic maturation-resistant APCs that maintain their immunoregulatory function even after LPS stimulation. Moreover, transplantation of such tolAPC-enriched corneal allografts onto vascularized high-risk host beds leads to reduced host allosensitization, prolonging corneal transplant survival.

Treatment of graft recipients with nonspecific immunosuppressive agents such as corticosteroids often leads to numerous side effects including opportunistic infections, and in the case of corneal transplants, development of cataracts and glaucoma. These deleterious side effects can be circumvented by alloantigen-specific tolerance-inducing strategies as presented here. According to the previous studies, injection of *ex vivo*-generated donor-type APCs with regulatory properties has shown promising results in experimental models of transplantation including corneal allografting^15, 19, 21, 22^. However, translation of such strategies into clinical transplantation is limited due to their practicality^23^. Considering that donor tissues carry resident APCs, we hypothesized that treating donor corneas with the anti-inflammatory cytokines IL-10 and TGFβ1 induces generation of tolAPCs in the graft. As human donor corneas are routinely stored in medium for several days to two weeks before grafting, we added IL-10 and TGFβ1 to murine corneal buttons cultured in medium, resulting in generation of tolAPCs (Supplemental Fig. 1A and B). However, using *ex vivo* treatment, we observed swelling of the murine corneal stroma after 4 days of incubation causing them to become unsuitable for transplantation. These results are in accord with published data showing that the mouse cornea, unlike that of the human, is subject to significant swelling soon after being maintained in culture medium due to a less developed Bowman’s and Descemet’s membranes, as well as loose stromal collagen lamellae^24^. To circumvent this limitation, we adopted an *in vivo* approach whereby donor buttons receive immunomodulatory cytokines *in situ* via subconjunctival injection in living donor animals prior to procurement. We demonstrate that subconjunctival injection of IL-10 and TGFβ1 alters the phenotypic and functional characteristics of tissue residents APCs by rendering the corneal microenvironment more immunoregulatory and less pro-inflammatory.

We showed that transplantation of IL-10 and TGFβ1-treated corneas reduces corneal cell infiltration in hosts post-surgery compared to hosts transplanted with saline-treated corneas. In order to determine whether injection of cytokines causes infiltration of donor immune cells into the cornea even before transplantation, we assessed the frequencies of bone marrow-derived cells in the donor cornea before grafting after cytokines injection. We show that cytokine injection itself had no effect on the frequencies of CD45^+^ leukocytes and CD11c^+^cells in the donor cornea compared to saline-injected controls and naïve corneas (Supplemental Figs 1A and 2B). To rule out possible “retention” of injected IL-10 and TGFβ1 in the donor cornea at the time of transplantation and to exclude direct effects of these cytokines on allosensitization and graft survival, we measured levels of IL-10 and TGFβ1 in the corneal buttons 48 hours after their injection but before transplantation. Our results indicate similar protein levels of IL-10 or TGFβ1 in the cytokine- vs. saline-treated group (Supplemental Fig. 2C), suggesting that reduced alloimmunity cannot be attributed to retained cytokine(s) in the donor tissue.

We have previously demonstrated that activation and migration of IFN-γ-producing Teffs to the graft site play a major role in corneal allograft rejection, and suppression of the alloimmune response improves long-term maintenance of graft clarity^6^. Here, we observed decreased frequencies of IFN-γ-producing CD4^+^ T cells and diminished numbers of activated T cells through both the direct and indirect pathways of sensitization (Fig. 2), resulting in less infiltration of leukocytes and CD4^+^ T cells to the graft site (Fig. 3). In heart transplantation, graft-infiltrating DC-SIGN^+^-suppressive macrophages mediate transplant tolerance by inhibiting T cell proliferation and expansion of Tregs^15^. Similar to our data, these investigators have reported that IL-10 is crucial for the suppressive function of tolAPCs.

It has been established that the indirect pathway plays a critical role in low-risk corneal graft recipients with non-inflamed graft beds. However, we have previously demonstrated that hosts with an inflamed and vascularized graft bed, which are at high risk of rejecting their transplant, are sensitized through both the direct and indirect pathways of allosensitization^25^. Using the same model in this study we show a diminished sensitization of host T cells via both the direct and indirect pathways of allosensitization (Fig. 2B). This is in accord with our *in vitro* experiments in which we show that immature recipient APCs can also acquire a maturation-resistant phenotype when co-cultured with donor tolerogenic dendritic cells (tolDCs) (Fig. 1C). Thus, in the aggregate, these findings suggest that graft-derived tolAPCs may also be able to confer maturation resistance to host APCs, though the precise mechanisms behind this remain unclear, and will be the subject for future studies.

In conclusion, this study reports that treatment of donor tissue with the immunomodulatory cytokines IL-10 and TGFβ1 results in generation of tolerogenic APCs that promote transplant survival. This is a novel strategy that can potentially be adapted to human donor corneal storage protocols for high-risk corneal transplantation.

## Materials and Methods

### Animals

Eight- to 10-week-old male BALB/c (H-2^d^) and C57BL/6 (H-2^b^) mice were obtained from Charles River Laboratories, Wilmington, MA. Animals were kept in a pathogen-free environment at the Schepens Eye Research Institute Animal Care Facility. All animal experiments were approved by the Institutional Animal Care and Use Committee of the Schepens Eye Research Institute, and were conducted in accordance with the Association for Research in Vision and Ophthalmology (ARVO) statement for the Use of Animals in Ophthalmic and Visual Research. Mice were anesthetized for surgical procedures using intraperitoneal (i.p.) injections of 120 mg/kg Ketamine and 20 mg/kg Xylazine.

### *In vivo* treatment of donor eyes with IL-10 and TGFβ1

C57BL/6 mice received single subconjunctival injection of IL-10 (6 ng/µl) and TGFβ1 (6 ng/µl) in 10 µl saline or saline alone (control). Injections were carried out using Hamilton syringe and needles (Hamilton Company, Reno, NV) under general anesthesia. 48 hours after cytokine injection, corneal buttons were harvested and transplanted or stimulated overnight with LPS (100 ng/ml) for flow cytometry and real-time PCR analyses.

### Orthotopic corneal transplantation onto inflamed high-risk graft beds

Corneal transplantation was performed using C57BL/6 corneal grafts and BALB/c hosts with inflamed graft beds as described previously^26^. Two weeks before transplantation three interrupted 8-shaped sutures (11-0 nylon, 50 µm diameter needle; Sharpoint; Angiotech, Vancouver, BC, Canada) were placed in the corneas of BALB/c hosts to induce inflamed and neovascularized graft beds. The central cornea of donor C57BL/6 mice was marked with a 2 mm diameter trephine and excised with Vannas scissors (Storz Instruments Company, San Damis, CA) and transplanted into a similarly prepared host bed of 1.5 mm diameter on the right eye of recipient (BALB/c) mice with 8 interrupted 11-0 nylon sutures (Sharpoint; Vanguard, Houston, TX). Eyelids were closed immediately after the surgery using 8-0 sutures and kept closed for two days. Corneal sutures were removed 7 days post-transplantation.

### Evaluation of graft survival

Transplanted corneas were examined weekly in a blinded fashion for 8 weeks (or until sacrificed for analyses) using a slit-lamp microscope. A standardized opacity grading system was used^27^; grafts with opacity scores of >2 (i.e. a level of opacity that obscures recognition of iris details) for at least two consecutive weeks at week 2 post-transplantation and onwards were considered as immune-rejected. Eyes that underwent complications during or after surgery including intraoperative hemorrhage, cataract, infection, or synechia as well as grafts that became opaque in the first two weeks after transplantation and never became clear were excluded from the analysis.

### *Ex vivo* treatment of corneal buttons with IL-10 and TGFβ1

Murine corneal buttons (2 mm in diameter) from C57BL/6 mice were harvested, placed in 96-well plates and incubated at 37 °C in RPMI (containing %10 FCS, penicillin, streptomycin and HEPES) with 20 ng/ml GM-CSF for cell survival during culture. IL-10 (20 ng/ml) and TGFβ1 (20 ng/ml), from Biolegend (San Diego, CA) were added to the media to induce maturation-resistant APCs in the cornea. After 4 days of incubation, corneas were treated with 100 ng/ml LPS (Sigma Aldrich, St. Louis, MO) overnight to mimic the inflammatory environment and induce maturation of resident APCs through TLR-4 stimulation. Corneas were then either immunostained or digested for flow cytometric analysis.

### Enzyme-linked immunosorbent assay

Levels of IL-10 and TGF-β1 in supernatants of corneal lysates were analyzed using commercially available murine ELISA kits per manufacturer’s instructions: Mouse IL-10 Single Analyte ELISArray Kit SEM03017A and Mouse TGFβ1 Single Analyte ELISArray Kit SEM02991A (Qiagen, Valencia, CA).

### Immunohistochemistry and Histology

Corneal buttons were de-epithelialized by 45 min incubation at 37 °C in EDTA (0.5 M in H_2_O, Sigma-Aldrich, Saint Louis, MO, diluted 25 times in PBS) and were fixed in acetone for 15 min at room temperature, followed by a rinse in Phosphate Buffer Saline (PBS). To prevent nonspecific staining, Fc receptor blocking antibody (anti-mouse CD16/CD32 purified, clone 93, eBioscience San Diego, CA) was used to block sections before staining. Corneas were incubated with antibodies at room temperature for 30–45 min. The following antibodies were used: Anti-Ia/Ib (MHC class II) (AF6-120.1), anti-CD45 (5C3), anti-CD86 (GL-1) from Biolegend (San Diego, CA) to stain corneal stromal APCs. Each step was followed by three thorough washings in PBS for 5–10 min. At the end samples were covered with mounting medium (Vector Laboratories) and analyzed by confocal laser scanning microscope (TCS 4D; Leica, Heidelberg, Germany). For histological evaluation, corneal sections were stained with H&E and examined using bright-filed microscopy.

### Cornea digestion and lymph node cell preparation

Corneal tissues were digested in RPMI media (Lonza, Walkersville, MD) containing 2 mg/ml collagenase type IV (Sigma-Aldrich, St. Louis, MO) and 2 mg/ml DNase I (Roche, Basel, Switzerland) for 60 min at 37 °C, and then filtered through a 70-µm cell strainer. Submandibular and cervical (draining) lymph nodes were harvested and a single cell suspension was prepared.

### Flow cytometry

Single cell suspensions were incubated with an Fc receptor blocking antibody before they were stained with the following antibodies: anti-CD45 (30-F11), anti-CD25 (PC61.5), and anti-Foxp3 (FJK-16s) from eBioscience (San Diego, CA); and anti-CD4 (RM4-5) and anti-IFN-γ (XMG1.2), anti-CD86 (GL-1), anti-CD40 (3/23), anti-Ia/Ie (M5/114.15.2) from Biolegend (San Diego, CA). In order to stain intracellular IFN-γ expression, cells were stimulated with phorbol 12-myristate 13-acetate (PMA; 50 ng/ml; Sigma-Aldrich) and Ionomycin (500 ng/ml; Sigma-Aldrich) for 6 hours in the presence of Golgistop (0.7 µl/100 µl media; BD Biosciences, San Jose, CA). For intracellular staining of IFN-γ and intranuclear staining of Foxp3, cells were fixed and permeabilized with appropriate buffers (eBioscience). Isotype controls were used for all antibodies. Cells were analyzed using the LSRII flow cytometer (BD Biosciences, Franklin Lakes, NJ) and Summit v4.3 Software (DAKO Colorado Inc., Fort Collins, CO).

### Cell Sorting

CD4^+^ T cells were isolated by magnetic separation from lymph nodes of naïve and transplanted BALB/c mice using CD4^+^ T cell Isolation kit (Miltenyi Biotec, Bergisch-Gladbach, Germany). The purity of isolated CD4^+^ T cells was >95% in all three groups as measured by flow cytometry. APCs were obtained by depleting T cells (CD90.2^+^ cells) from splenocytes of C56BL/6 mice using a CD90.2 isolation kit (Miltenyi Biotec).

### ELISPOT assay

ELISPOT assay was used to analyze the numbers of IFNγ-producing T cells allosensitized by the direct and indirect pathway, as described previously^19^. In brief, 96-well ELISPOT plates (Multiscreen, Darmstadt, Germany) were coated with anti-IFN- antibody (BD Pharmingen, San Diego, CA), and blocked with 1% BSA. Purified T cells from allografted BALB/c mice two weeks post-transplantation (250,000 CD4^+^ T cells, MACS-sorted) were incubated with (1) C57BL/6 APCs (500,000 CD90.2^−^, MACS sorted splenocytes) to quantify frequencies of directly allosensitized T cells or (2) with syngeneic APCs pulsed with sonicated donor antigen (from 10^6^ cell/50 µl single cell suspension of C57BL/6 spleen) to quantify frequencies of indirectly allosensitized T cells. T cells harvested from LNs of naive BALB/c animals served as controls. After 48 hours of incubation, plates were washed and a biotinylated anti-IFN-γ detection mAb was added at 2 μg/ml (BD Pharmingen, San Jose, CA) and incubated overnight at 4 °C. The plates were then washed, incubated for 1 hour with Avidin-HRP and developed using aminoethylcarbazole staining solution (Sigma-Aldrich, Springfield, MO). The resulting spots were analyzed using the computer-assisted ELISPOT image analyzer (Cellular Technology Ltd., Cleveland, OH).

### Real-time PCR

Using Trizol (Ambion, Life Technologies, Grand Island, NY) tissues were homogenized on ice and RNA was precipitated in the aqueous phase using 70% ethanol, followed by subsequent extraction and purification using RNeasy Micro kit (Qiagen, Valencia, CA, USA). Reverse transcription of total RNA was conducted using the Superscript III kit (Invitrogen, Carlsbad, CA, USA). Real-time PCR was performed using TaqMan Universal PCR Mastermix (Applied Biosystems, Foster City, CA, USA) and preformulated primers for IL-10 (Mm00439614_m1), TNF-α (Mm00443258_m1) and GAPDH (Mm99999915_gl). The PCR conditions were 40 cycles at 95 °C for 30 s, 58 °C for 30 s, and 72 °C for 1 min, followed by final extension at 72 °C for 10 min. The results were analyzed by the comparative threshold cycle method and normalized to GAPDH as an internal control.

### Statistical analyses

Student’s t test was used to compare two groups. P values of < 0.05 were considered statistically significant. The Kaplan-Meier survival curve was used to determine graft survival and Wilcoxon Rank test was used to compare survival rates between the groups.

## Electronic supplementary material

Supplementary Figures
