# Supplementary material for: Treatment of donor corneal tissue with immunomodulatory cytokines: a novel strategy to promote graft survival in high-risk corneal transplantation
Source: Sci Rep. 2017 Apr 20;7:971. doi: 10.1038/s41598-017-01065-z (PMC5430534; doi:10.1038/s41598-017-01065-z)
Supplement: Supplementary file 1 — Supplementary Figures [file 41598_2017_1065_MOESM1_ESM.doc]

**Treatment of donor corneal tissue with immunomodulatory cytokines: a novel strategy to promote graft survival in high-risk corneal transplantation**

Maryam Tahvildari, MD^1^; Parisa Emami-Naeini, MD, MPH^1^; Masahiro Omoto, MD^1^; Alireza Mashaghi, PhD^1^; Sunil K. Chauhan, MD, DVM^1^; Reza Dana, MD, MSC, MPH^1,*^

**Supplementary Information**

**Supplemental Figure 1. Ex vivo manipulation of donor corneas with IL-10 and TGFβ results in generation of tolerogenic APCs.** Donor corneal buttons (2 mm in diameter) were harvested and incubated at 37˚C in RPMI containing IL-10 (20 ng/ml), TGFβ1 (20 ng/ml), and GM-CSF (20ng/ml) or RPMI containing GM-CSF only (control; GM-CSF was added to maintain survival). 4 days after incubation, corneas were treated with LPS (100 ng/ml) overnight, then de-epithelialized and either immunostained or digested for flow cytometry. (A) Immunohistochemistry and (B) flow cytometry show increased expression of MHCII and CD86 by resident CD45^+^ cells in the corneal stroma of control corneas compared to fresh corneas. IL-10/TGFβ1-treated corneas showed very low frequencies of MHC^+^ or CD86^+^ CD45^+^ cells, similar to fresh corneas (N=10 corneas/group).

**Supplemental Figure 2. Effect of subconjunctival injection of IL-10 + TGFβ1 or saline on CD45^+^ and CD11c^+^ cell infiltration and cytokine levels in the donor cornea.** Donor eyes received a single subconjunctival injection of either IL-10 + TGFβ1, or saline (as control). 48h later, 2mm central corneas were harvested. Flow cytometry analysis showing frequencies of (A) CD45^+^ and (B) CD11c^+^ cells in the donor cornea 48 hours after injection of IL-10 + TGFβ1 or saline, indicating that subconjunctival injection alone does not increase infiltration of leukocytes into the graft. (C) ELISA of homogenized corneal tissue revealed similar levels of IL-10 and TGFβ1 in the IL-10/TGFβ1- and saline-treated corneas, indicating that these cytokines are not retained within the corneal tissue 48 hours after subconjunctival injection (N=6 corneas/group).
